# Fecal microbial composition and functional diversity of Wuzhishan pigs at different growth stages

**DOI:** 10.1186/s13568-021-01249-x

**Published:** 2021-06-12

**Authors:** Mingying Shao, Zhixin Wang, Yingzhi He, Zhen Tan, Jibin Zhang

**Affiliations:** 1 State Key Laboratory of Agricultural Microbiology, College of Life Science and Technology, Huazhong Agricultural University, Wuhan, 430070 China; 2 College of Tropical Agriculture and Technology, Hainan College of Vocation and Technique, Haikou, 570216 China; 3 College of Animal Science and Technology, Hainan University, Haikou, 570228 China

**Keywords:** Wuzhishan pig, Fecal microbiota, 16S rDNA, Growth stages

## Abstract

The mammalian gut microbiome participates in almost all life processes in the host. In addition to diet, the breed is the main factor affecting changes in the swine gut microbiota. The composition of the gut microbiota changes significantly during different growth stages. Research on developmental changes in the gut microbiota of indigenous Chinese pig breeds is limited. In this study, the fecal microbiota of Wuzhishan pigs (a Chinese indigenous miniature pig) at different growth stages was investigated using high-throughput 16S rRNA sequencing. *Firmicutes* and *Bacteroidetes* were the two dominant phyla, accounting for more than 80% of all sequences. With increasing age, the fecal microbial diversity increased, and the proportion of *Firmicutes* increased, whereas the proportion of *Bacteroidetes* decreased. A total of 49 biomarkers with statistical differences were detected in the four growth stages. The different microbiota among groups enhanced the ability to degrade fiber, carbohydrates, and other substances during the growth stages. The endocrine system was different in multiple growth stage paired comparisons, which was attributed to the different body statuses in the growth stages. This study revealed developmental changes in the structure and function of gut microbes in local pigs.

**Supplementary Information:**

The online version contains supplementary material available at 10.1186/s13568-021-01249-x.

## Introduction

From birth to market, the gut microbiome of pigs during the entire growth period is not immutable, but rather a dynamic development process. A high degree of microbial diversity has been observed in the meconium of newborn piglets, with Bacteroides, Firmicutes, Proteobacteria, and Fusobacteria dominating at birth. Despite their low concentrations, these microorganisms may act as stains to promote the development of the pig gut microbiome during the subsequent growth stages. After birth, piglets develop a simple microbial environment in their gastrointestinal tract, which is affected by the maternal birth canal, feces, and surrounding environment microorganisms. Over time, anaerobes inhibit the proliferation of aerobes and facultative aerobes and gradually dominate the colonizing bacteria (Heinritz et al. 2013; Leser et al. 2002). During the lactation period, bacteria such as *Lactobacillus* and *Streptococcus* can more effectively use the nutrients in breast milk; therefore, this type of bacteria has an advantage in the stomach and small intestine of piglets (Derrien and Vlieg 2015). On the first day of birth, various bacteria begin to colonize the intestinal segments of piglets. After 1 to 3 weeks, *bifidobacteria*, *Escherichia coli*, *Lactobacillus, Peptococcus*, and *Bacteroides* gradually increase and become the dominant bacteria in the intestinal tract (Ducluzeau 1983). Before weaning, the microbial community structure dominated by anaerobic bacteria is very unstable. Weaning isolation, feed, and living environment changes can quickly disrupt the balance of the original intestinal microbial community and gradually form a new state of balance of the microbial community structure. The drastic changes in the intestinal microbiota under various stimuli gradually return to a normal and stable state of balance as the interfering factors are eliminated (Dethlefsen et al. 2008; Melin et al. 2004).

*Firmicutes* and *Bacteroidetes* are the dominant bacteria in the gut microbes of pigs before and after weaning, accounting for more than 90% of all bacteria by 16S rDNA sequencing (Kim et al. 2011). At the genus level, the proportion of *Bacteroides* from the higher abundance before weaning was significantly reduced after weaning, and *Prevotella* and *Clostridium* became the dominant genera (Pajarillo et al. 2014a).

Gut microbes are also influenced by pig genotypes. A high abundance of *Firmicutes* and a low abundance of *Bacteroidetes* are found in the intestines of obese pigs (Guo et al. 2008), and the number of specific microbes varies among different pig breeds. With increasing age, the number and abundance of dominant bacteria change. Su et al. confirmed that different pig breeds and growth stages influenced intestinal microorganisms; however, there was no significant difference in intestinal microorganisms among different pig breeds, while different growth stages influenced the intestinal microbial composition (Su et al. 2014). By studying the differences in gut microbes in eight pig breeds, Yang et al. distinguished the types of pig gut microbes. Among them, the intestinal microbes of foreign breeds of Landrace, Yorkshire, and Duroc had a high similarity, whereas some breeds of local pigs, Bama pigs, Erhualian pigs, and a few Meishan pigs had high similarity in their gut microbial structure, and some unique microorganisms were found in Chinese local breeds (Yang et al. 2014).

Compared with foreign pig breeds, Chinese local pig breeds have disadvantages of slow growth rate, low lean meat rate, long feeding cycle, and high cost. However, local pig breeds have the unique advantages of a high reproduction rate, strong disease resistance, and good meat quality. The Wuzhishan minipig is a special pig in China, initially grazing in isolated tropical areas in Hainan, and is one of the distinctive local pig genetic resources in China. It has a good meat quality, resistance to coarse feeding and high temperature, and is an ideal animal model for scientific research. The growth conditions of Wuzhishan pigs are close to Southeast Asia, hot and humid climate, lush vegetation, prompting the pigs have differences with intensive commercial species in physical and life mode. Research on the germplasm characteristics of Wuzhishan pigs has not received enough attention in China, mostly focusing on genetic inheritance and cloning. Understanding the characteristics of local pigs can help to fully explore the genetic resources of local pigs, give full attention to their genetic potential, and implement the development of local pig products to improve the utilization rate, increase the economic breeding benefits, and promote the conservation, development, and utilization of local pigs. In the present study, we explored the growth stage differences in the composition and function of fecal microorganisms in Wuzhishan pigs using 16S rRNA gene sequencing. Our findings provide an important reference for more in-depth studies on the complex interactions between the microbiome and indigenous pigs.

## Materials and methods

### Experimental animals and sample collection

All Wuzhishan pigs were collected from a commercial farm in Wenchang, Hainan Province, China. The animals were housed in a semi-enclosed room with an ambient temperature of 25–35 °C. Fresh fecal samples were collected from individual pigs in four growth stages: pre-weaning (PW, 2.5–3.5 kg, n = 4), weaning piglet (WP, 3.5–4.5 kg, n = 6), growing pig (GP, 7–10 kg, n = 4), and sow (SP, 20–26 kg, n = 7). The pigs were provided with a commercial diet with ad libitum access to cleanwater. All animals were healthy and did not receive any antibiotics during the study. Each fresh fecal sample was collected randomly from each pen and stored in a 2 mL centrifuge tube and kept on ice during transportation. All samples were stored in a − 80 °C freezer for cryopreservation until DNA extraction.

### DNA extraction and polymerase chain reaction (PCR) amplification

Fecal microbial genomic DNA was extracted using the QIAamp DNA Stool Mini Kit (Qiagen Ltd., Hilden, Germany), following the manufacturer’s standard protocol. The V3-V4 region of the bacterial 16S rDNA was PCR-amplified using the forward primer 338F (5′-ACTCCTACGGGAGGCAGCA-3′) and reverse primer 806R (5′-GGACTACHVGGGTWTCTAAT-3′). The total reaction volume was 30 µL and the GeneJET Gel Extraction Kit (Thermo Fisher Scientific, Waltham, MA, USA) was used to purify the PCR products following the manufacturer’s protocol. The constructed library was sequenced on an Illumina HiSeq 2500 platform (2 × 250 paired ends).

### 16S rRNA gene sequence assembly and clustering

Sequencing was performed by Biomarker Technologies Corporation (Beijing, China). After merged using FLASH version 1.2.7 (Magoc and Salzberg 2011) and quality controlled using Trimmomatic version 0.33 and UCHIME version 4.2 (Bolger et al. 2014; Edgar et al. 2011), sequences with at least 97% similarity to obtain operational taxonomic units (OTUs) were clustered using USEARCH version 10.0 (Edgar 2013). OTUs with a threshold of 0.005% of the total sequences were filtered out. Representative sequences of OTUs were screened and taxonomically analyzed against the 16S rRNA microbial reference database Silva, using the Ribosomal Database Project Classifier version 2.2 (Quast et al. 2013). The relative abundance of each OTU was assessed in each sample by counting the number of tags. Community composition was assessed at the phylum and genus levels using the QIIME version 1.8.0 (Caporaso et al. 2010).

### Diversity analysis and functional predictions

Mothur version v.1.30 (Schloss et al. 2009) was used to evaluate alpha diversity indices. QIIME software (version 1.8.0) was used to calculate beta diversity by principal coordinate analysis based on weighted UniFrac distances (Lozupone et al. 2011; Segata et al. 2011). The line discriminant analysis effect size (LEfSe) was used to identify biomarkers with significant differences in abundance among different groups (Looft et al. 2012; Segata et al. 2011). Functional predictions were performed using PICRUSt (Langille et al. 2013).

## Results

### OTU clustering and taxonomy analysis

After quality filtering, we obtained 1,474,150 effective reads from 21 fecal samples with an average length of 417 base pairs. Each sample contained approximately 70 000 sequences on average (Additional file 1: Table S1), ranging from 31,732 to 56,822 reads. A total of 708 OTUs were detected, of which 569 were shared among all groups (Additional file 2: Fig. S1). The Chao1 index of the PW group was lower than that of the WP and GP groups. The GP group had significantly higher alpha indices than the PW group. The Simpson index was higher in the SP group than in the PW group (Additional file 3: Fig. S2).

**Fig. 1:**
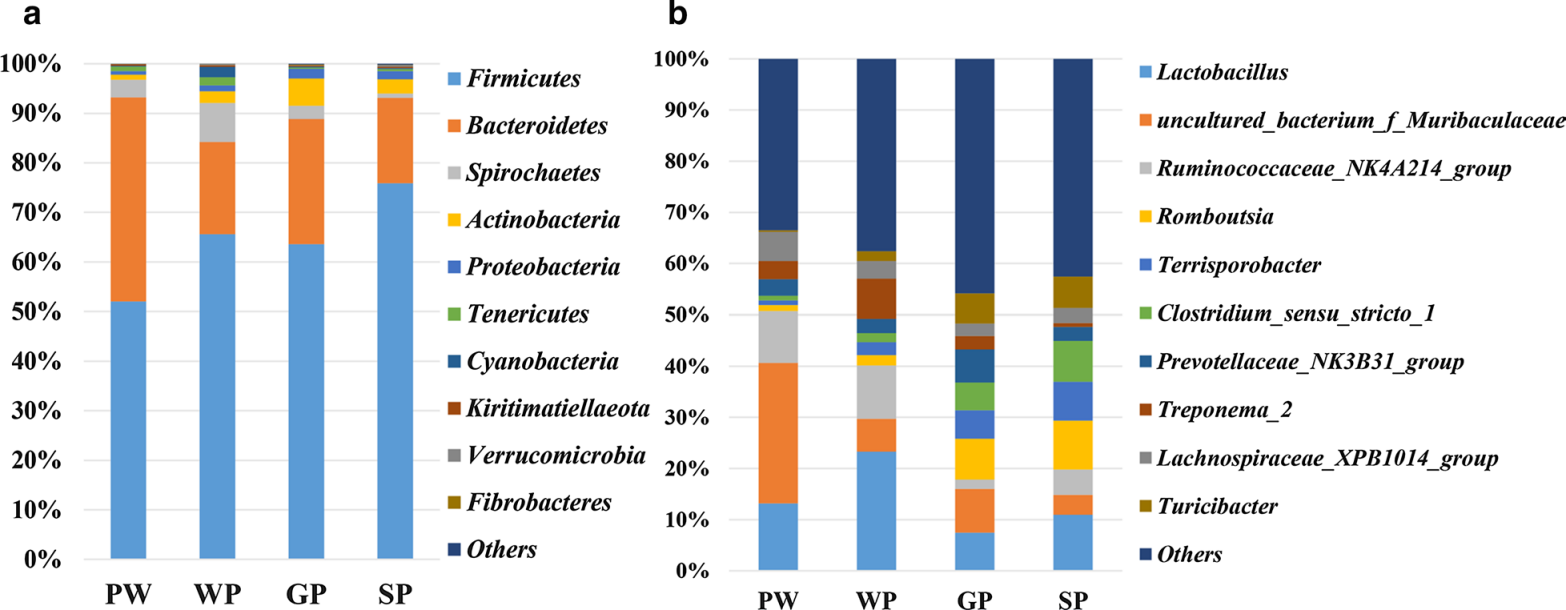
Histogram of the top 10 phylum (**A**) and genus (**B**) in each group

*Firmicutes* and *Bacteroidetes* were the two dominant phyla, accounting for more than 80% of all sequences in each group (Fig. 1A). The proportion of Firmicutes in the PW group was 51.98% and 75.85% in the SP group, whereas the proportion of *Bacteroidetes* was 41.24% in the PW group and 17.28% in the SP group. The relative abundance of *Spirochaetes* was 7.85% in the WP group and 3.55%, 2.65%, and 0.84% in the PW, GP, and SP groups, respectively. *Actinobacteria* accounted for 5.54% in the GP group and no more than 3% in the other three groups.

At the genus level, the proportion of *Lactobacillus* was 13.18%, 23.29%, 7.46%, and 10.95% in the PW, WP, GP, and SP groups, respectively (Fig. 1B). *Uncultured_bacterium_f_Muribaculaceae* (*S24-7*) in the PW group was 27.46%, whereas it was 6.43%, 8.55%, and 3.90% in the WP, GP, and SP groups, respectively. The proportion of *Ruminococcaceae_NK4A214_group* was more than 10% in the PW (10.08%) and WP (10.42%) groups, and less than 5% in the GP (1.80%) and SP (4.96%) groups. The relative abundances of *Romboutsia* accounted for 1.16% in the PW and 2.02% in the WP groups, and 8.00% in the GP and 9.57% in the SP groups.

### Comparison of fecal microorganisms among the four pig groups

The principal coordinate analysis revealed that samples from the different growth stages showed more distant separation, whereas samples from the same group were more similar (Additional file 4: Fig. S3). Samples in the PW group were clustered and far away from the other three groups, indicating that the gut microbial structure of PW piglets was significantly different from that of the other stages. While the samples within the groups were close to each other, indicating that these samples were similar (Additional file 5: Fig. S4).

**Fig. 2:**
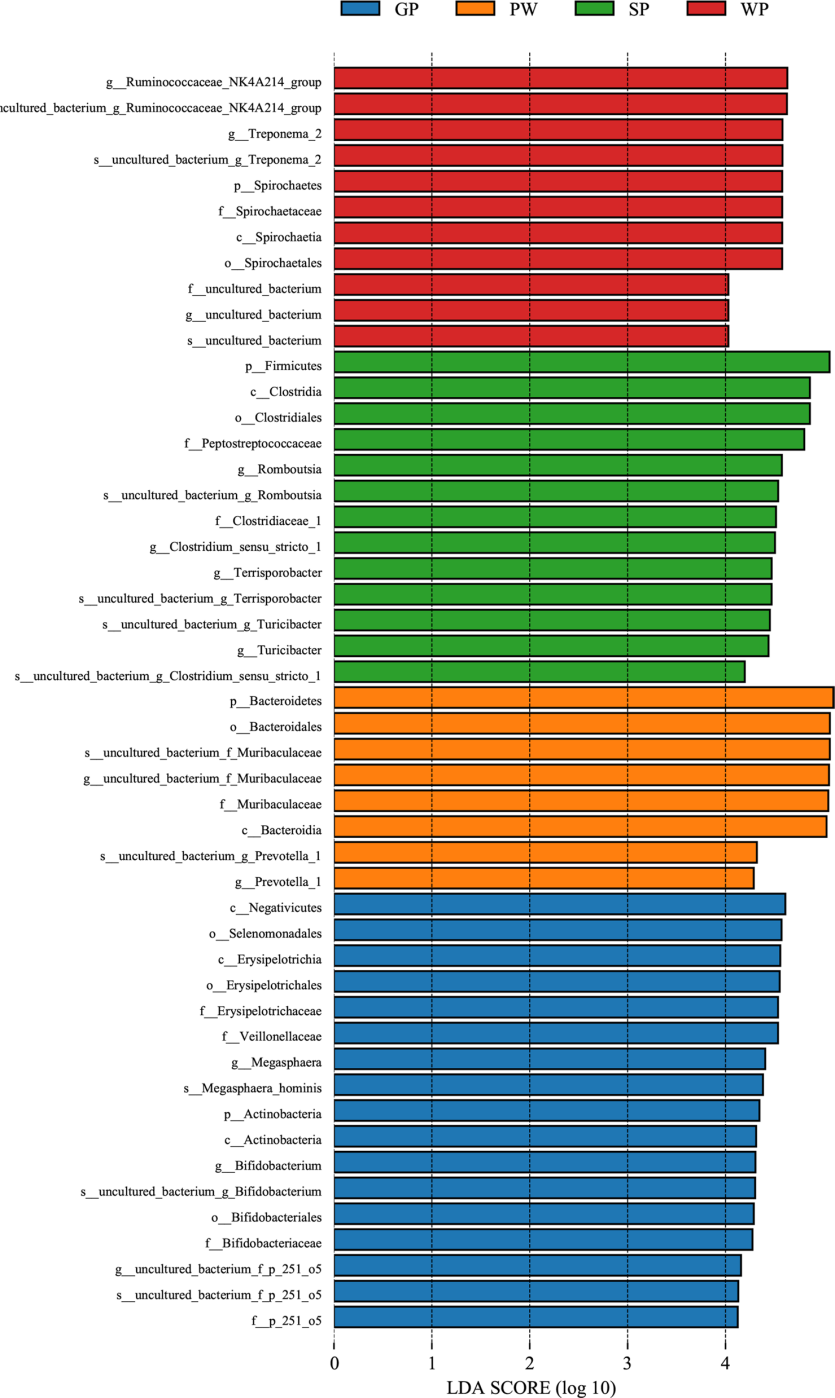
Fecal microbiota phylotypes differ of pigs at various grow stages. **A** Histogram of linear discriminant analysis (LDA) scores computed for differences in the proportions of fecal microbiota. Taxa meeting an LDA significant threshold of > 4 are shown

A total of 49 biomarkers with statistical differences were detected using LEfSe (8 in the PW, 11 in the WP, 17 in the GP, and 13 in the SP groups) in Fig. 2. *Bacteroidetes* was significantly enriched in the PW group, *Spirochaetes* was enriched in the WP group, the relative abundance of *Actinobacteria* was highest in the GP group, and the SP group was significantly enriched in the phylum *Firmicutes*. A total of 11 genera could be potential biomarkers to distinguish the growth stages of pigs. The genus *Prevotella_1* was significantly enriched in the PW group, *Treponema_2* and *Ruminococcaceae_NK4A214_group* were enriched in the WP group, whereas the GP group was significantly enriched in the genera *Megasphaera* and *Bifidobacterium*, and the relative abundances of the genera *Romboutsia*, *Terrisporobacter*, and *Turicibacter* were most abundant in the SP group. These significantly different abundances of bacteria were consistent with the taxonomic analysis described above.

### Differences of microbial function among the four pig groups

**Fig. 3:**
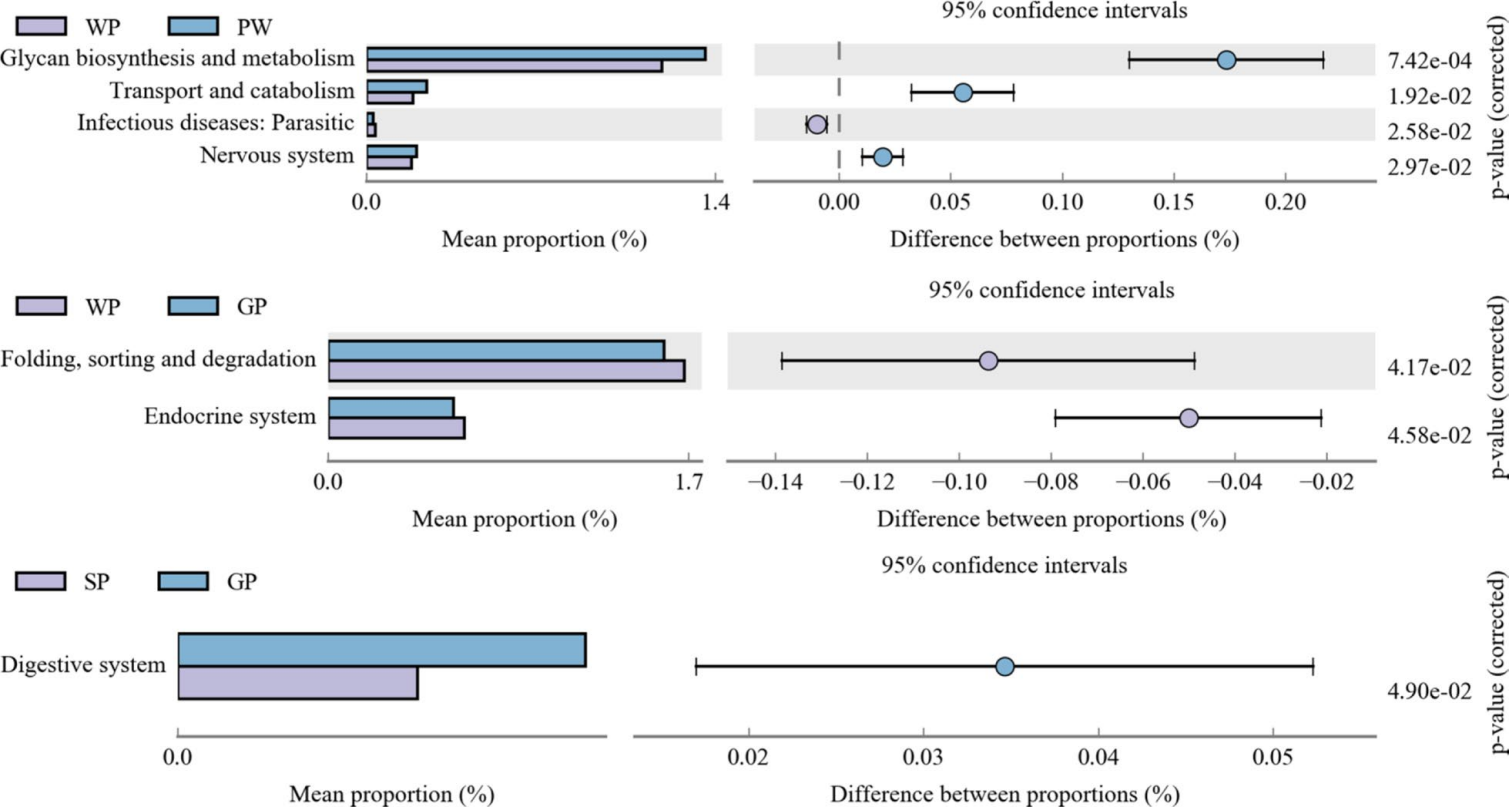
Comparison of enriched KEGG metabolic pathways in fecal microbiota of pigs at different grow stages. The left figure in the picture shows the abundance ratio of different functions in two groups of samples, the middle shows the difference ratio of functional abundance in the 95% confidence interval, and the right value is the p‐value. KEGG, Kyoto Encyclopedia of Genes and Genomes

A total of 46 KEGG metabolic pathways were detected in class two. Four groups were compared in pairs, and 24 metabolic pathways were significantly different in all six pairs (Fig. 3, Additional file 6: Fig. S5).

Microbial functional differences between two adjacent growth stages were compared. Four pathways were significantly different between PW and WP, except pathway infection diseases: parasitic, and the other three pathways glycan biosynthesis and metabolism, transport and catabolism, and nervous system were all significantly enriched in the PW group. Folding, sorting, degradation, and the endocrine system were significantly enriched in WP compared with the GP group. The abundance of the digestive system in the GP group was higher than that in SP (Fig. 3). Comparison of other pairs revealed that 12 metabolic pathways were different between the PW and GP groups, including carbohydrate metabolism, metabolism of cofactors and vitamins, folding, sorting and degradation, and cellular community-prokaryotes. Metabolism of cofactors and vitamins, transport and catabolism, membrane transport, and endocrine system were different between the PW and SP groups. In addition, the endocrine pathway was different between the WP and SP groups (Additional file 6: Fig. S5).

**Fig. 4:**
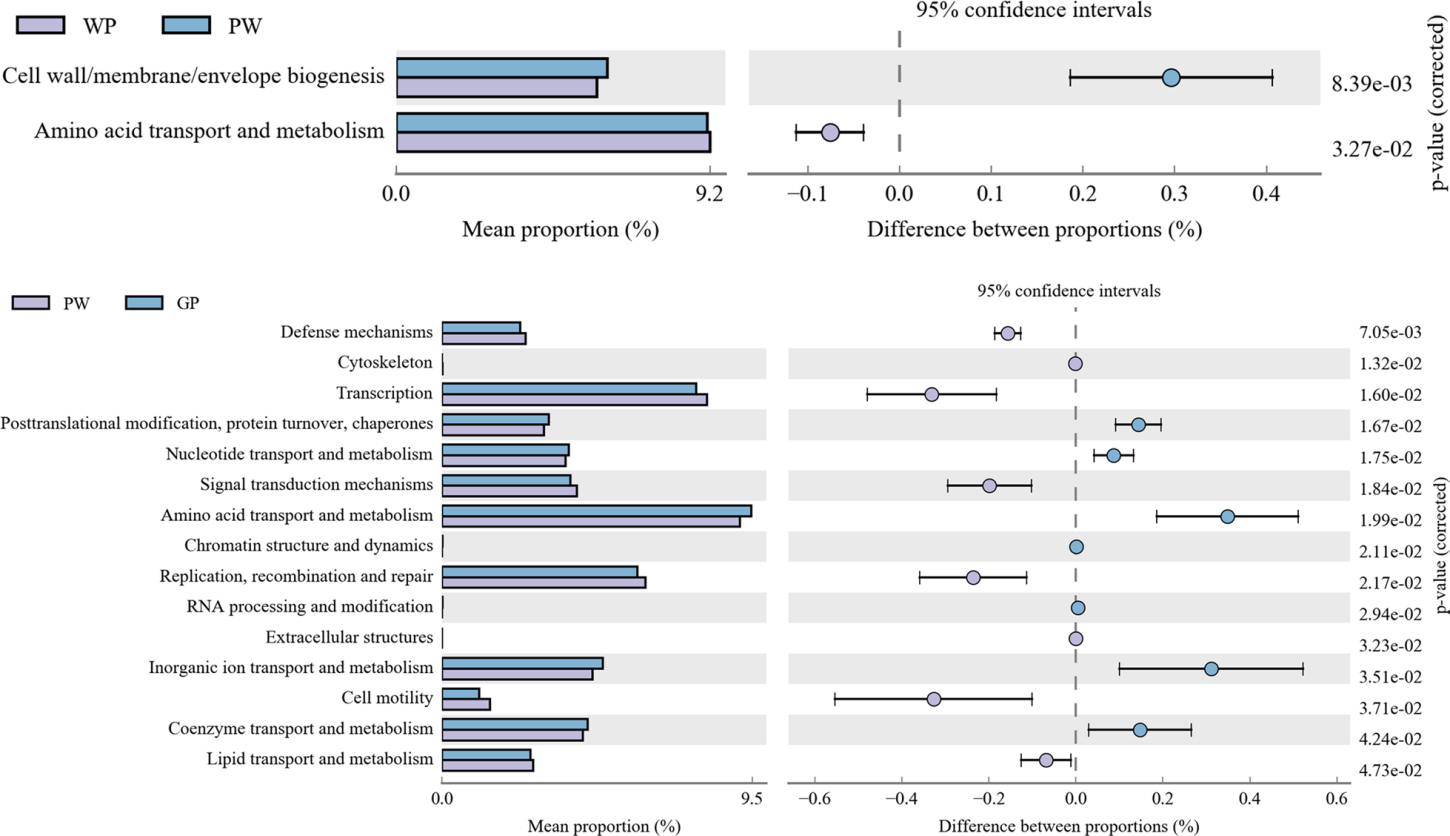
Comparison of COG functions in fecal microbiota of pigs at different grow stages. Proportion of functional abundance differences within the 95% confidence interval. COG, Clusters of Orthologous Groups of proteins

Compared with COG, a total of 25 s-level classifications were annotated. Cell wall/membrane/envelope biogenesis and amino acid transport and metabolism enrichment differed between the PW and WP groups. A total of 15 annotations were significantly different between the PW and GP groups, including amino acid transport and metabolism, transcription, replication, recombination and repair, inorganic ion transport and metabolism (Fig. 4). No significant differences were found in other pairwise comparisons.

## Discussion

Pigs are important animal models for human diseases (Niu et al. 2015), especially mini breeds. The Wuzhishan pig is a famous miniature pig breed in China. A highly inbred line is an ideal animal model for medical experiments. The distribution of the gut microbiota across the pig growth stages has been studied previously (Kim et al. 2011, 2012, 2015). Both bacterial abundance and diversity increased with growth stages, and during the weaning period, the pig fecal microbiota shifted, causing a physiologically stressful time for animals (Pajarillo et al. 2014b). After weaning, the composition of the gut microbiota continued to change until market age (Kim et al. 2011). Breeds also have an important effect on the structure of gut microbiota. Several bacterial taxa differentially exist within specific intestinal segments in Landrace and Jinhua pigs, a Chinese indigenous slow-growing breed with a high propensity for fat deposition. Functional prediction of microorganisms showed that the fatty acid biosynthesis pathway of Jinghua pigs was higher than that of Landrace pigs, partially explaining their adiposity phenotype (Xiao et al. 2018). Compared with the obese Erhualian pigs, the diversity and density of methanogens in the feces of lean-type Landrace pigs were higher (Luo et al. 2012). The goal of the present study was to investigate the characteristics of the gut microbiota in different growth stages, including pre-weaning and weaning piglets, growing pigs, and sows of the Wuzhishan miniature breed, to elucidate the changes in the microbial structure and its correlation with age, and the gut microbial specificity of indigenous miniature pigs.

The Chao1 index explored bacterial abundance, and the Simpson and Shannon indices explored bacterial diversity. The microbial complexity before weaning was significantly lower than during the grower stage, which was consistent with previous studies using other pig breeds (Kim et al. 2011; Mach et al. 2015; Niu et al. 2019, 2015). *Firmicutes* and *Bacteroidetes* were the two dominant phyla, representing more than 80% of all sequences in each group. With increasing age, the proportion of *Firmicutes* increased, whereas the proportion of *Bacteroidetes* decreased. Similar results were observed in pig and human fecal samples in previous studies (Isaacson and Kim 2012; Kim et al. 2011, 2015). The LEfSe analysis showed that *Spirochaetes* had significant variation in the feces of the WP group, whereas *Actinobacteria* was enriched in the GP group. Many species of *Treponema*, belonging to *Spirochaetes*, have been reported to be pathogenic bacteria (Stamm et al. 2009), and the proportion of the genus *Treponema* in feces might indirectly reflect the health status of animals. Because of weaning stress and environmental changes, the proportion of pathogenic bacteria in weaned pigs may increase. At the genus level, the structures of dominant genera in the GP and SP groups were different before and after weaning. The proportion of *Romboutsia* and *Turicibacter* increased, which was linked to fat accumulation (Guo et al. 2018) and butyric acid (Yin et al. 2018). The present study and previous research showed discrepancies, which may be due to the use of pigs of different ages, environmental conditions, or breeds (Niu et al. 2019).

Through the comparison of KEGG pathways, compared with the WP group, the fecal microorganisms of the PW group showed functional enrichment in glycan biosynthesis and metabolism, transport and catabolism, and the nervous system. During this period, the living environment of piglets changes dramatically, the structure of the gut microbiota changes accordingly, and the functional pathways related to substance digestion and intestinal health may appear to be different. The WP group had more enriched pathways related to folding, sorting, degradation, and the endocrine system than the GP group. The endocrine system pathway was also different in other paired comparisons, with enrichment in fecal microbiota of pre-weaning and post-weaning piglets than in sows. Endocrine regulation changes dramatically with age; therefore, microorganisms may also vary in their corresponding metabolic pathways. The GP and SP groups had different digestive systems. Compared to the SP group, the GP group was not fully mature in their physical development. The gut microbiota may assist the digestive system in digesting certain amounts of material.

Previous studies have found that the abundance of *Clostridium* and *Turicibacter* was positively correlated with apparent ether extract digestibility, whereas *Anaerofustis* and *Robinsoniella* were positively correlated with apparent crude fiber digestibility in sow fecal samples (Niu et al. 2019, 2015). Pigs at different growth stages have different abilities to digest materials, and the difference in microorganisms is closely related to the digestibility of materials. These differences reveal the differences in the regulation of basic functions of intestinal microorganisms and could signal differences between growth stages in gut function.

Future research should examine more closely at the characteristics of gut microbial composition of local pigs at various growth stages, and the production efficiency of animals by regulating the gut microbiome, which would produce a more comprehensive understanding of the breed characteristics and rational utilization of local pigs.

The present study showed clear differences in the gut microbiomes of various growth stages of Wuzhishan pigs. With increasing age, the fecal microbial diversity increased and the proportion of *Firmicutes* increased, whereas the proportion of *Bacteroidetes* decreased. The basic microbial composition was similar to that of other breeds; however, the relative abundance was specific. The different microbiota among groups enhanced the ability to degrade fiber, carbohydrates, and other substances during growth stages. The endocrine system was different during the multiple growth stage paired comparison, which was attributed to the different body statuses during the growth stages. The present study revealed the developmental changes in the structure and function of the gut microbes of local pigs and provided an important reference for future research on nutritional regulation by the gut microbiota.

## Supplementary Information

**Additional file 1: Table S1. **Statistics of sample sequencing data processing results.

**Additional file 2: Fig. S1.** Shared OTU analysis of the different groups. PW, fecal microbiota of the preweaning piglets; WP, fecal microbiota of the weaning piglets; GP, fecal microbiota of the growth pigs; SP, fecal microbiota of the sow.

**Additional file 3: Fig. S2.** Dynamic changes in intestinal microorganism alpha diversity. (A) Chao1 index; (B) Shannon index, (C) Simpson index. Different letters represent significant differences in alpha diversity indices based on Student’s *t*-test (* *p* < 0.05, ** *p* < 0.01).

**Additional file 4: Fig. S3.** Principal coordinates analysis (PCoA) in fecal microbiota of pigs at different grow stages.

**Additional file 5: Fig. S4.** Unweighted pair-group method with arithmetic mean (UPGMA) phylogenetic tree analysis.

**Additional file 6: Fig. S5.** Analysis of the difference of KEGG metabolic pathway between groups at the second level. Comparison between preweaning piglets and growth pigs, between preweaning piglets and sow, between weaning piglets and sow. The proportion of functional-abundance differences within the 95% confidence interval.

## Data Availability

The data have been deposited in the National Center for Biotechnology Information’s Short Read Archive under PRJNA736793.
