# Supplementary material for: Fecal microbial composition and functional diversity of Wuzhishan pigs at different growth stages
Source: AMB Express. 2021 Jun 12;11:88. doi: 10.1186/s13568-021-01249-x (PMC8197691; doi:10.1186/s13568-021-01249-x)
Supplement: Supplementary file 1 — Additional file 1: Table S1. Statistics of sample sequencing data processing results. [file 13568_2021_1249_MOESM1_ESM.docx]

**S1 Table. Statistics of sample sequencing data processing results.**

| Sample ID | Raw Reads | Clean Reads | Effective Reads | AvgLen(bp) | GC(%) | Q20(%) | Q30(%) | Effective(%) |
| --- | --- | --- | --- | --- | --- | --- | --- | --- |
| GP1 | 78848 | 77545 | 69920 | 417 | 53.45 | 98.19 | 94.44 | 88.68 |
| GP2 | 78634 | 77175 | 67894 | 417 | 53.68 | 98.07 | 94.24 | 86.34 |
| GP3 | 78799 | 77362 | 67542 | 418 | 54.15 | 98.06 | 94.24 | 85.71 |
| GP4 | 78748 | 77346 | 68219 | 418 | 54.08 | 98.13 | 94.35 | 86.63 |
| LF1 | 78667 | 77467 | 74720 | 416 | 53.07 | 98.16 | 94.45 | 94.98 |
| LF2 | 78393 | 76870 | 72865 | 416 | 53.33 | 98.08 | 94.2 | 92.95 |
| LF3 | 78386 | 76704 | 71600 | 420 | 52.74 | 98.02 | 94.02 | 91.34 |
| LF4 | 78551 | 76758 | 71320 | 420 | 52.67 | 97.9 | 93.87 | 90.79 |
| LF5 | 78903 | 77098 | 70609 | 419 | 52.7 | 97.96 | 93.96 | 89.49 |
| LF6 | 78970 | 77182 | 72131 | 420 | 52.52 | 97.92 | 93.91 | 91.34 |
| LM1 | 78663 | 76812 | 71755 | 418 | 53.85 | 97.93 | 93.94 | 91.22 |
| LM2 | 78272 | 76640 | 72429 | 417 | 53.61 | 97.95 | 94.01 | 92.54 |
| LM3 | 78637 | 77307 | 73166 | 418 | 53.7 | 98.1 | 94.25 | 93.04 |
| LM4 | 78311 | 76969 | 73290 | 417 | 53.65 | 98.02 | 94.12 | 93.59 |
| SP1 | 77925 | 76223 | 67658 | 414 | 53.43 | 97.95 | 94.06 | 86.82 |
| SP2 | 78280 | 76243 | 67975 | 420 | 53.27 | 98.08 | 94.2 | 86.84 |
| SP3 | 78303 | 76928 | 69769 | 415 | 53.36 | 98.39 | 94.82 | 89.1 |
| SP4 | 78474 | 76957 | 71197 | 414 | 52.98 | 98.21 | 94.48 | 90.73 |
| SP5 | 78701 | 77278 | 68089 | 417 | 53.19 | 98.26 | 94.56 | 86.52 |
| SP6 | 78568 | 77072 | 64960 | 416 | 53.15 | 98.27 | 94.59 | 82.68 |
| SP7 | 78689 | 76990 | 67042 | 415 | 53.47 | 98.18 | 94.42 | 85.2 |

Sample ID is sample name; Raw Reads is double-terminal reads splicing original sequence number; Clean Reads is original sequence number; Effective Reads is Clean Reads effective sequence number after filtering chimera; AvgLen (bp) is sample average sequence length; GC (%) percentage of bases of G and C types to total bases. Q20 (%) is the percentage of bases with mass value greater than or equal to 20 in total bases; Q30 (%) is the percentage of bases with mass value greater than or equal to 30 in total bases; Effective (%) is the percentage of Effective Reads in Raw Reads.
