# Supplementary figures and images for: Fecal microbial composition and functional diversity of Wuzhishan pigs at different growth stages
Source: AMB Express. 2021 Jun 12;11:88. doi: 10.1186/s13568-021-01249-x (PMC8197691; doi:10.1186/s13568-021-01249-x)

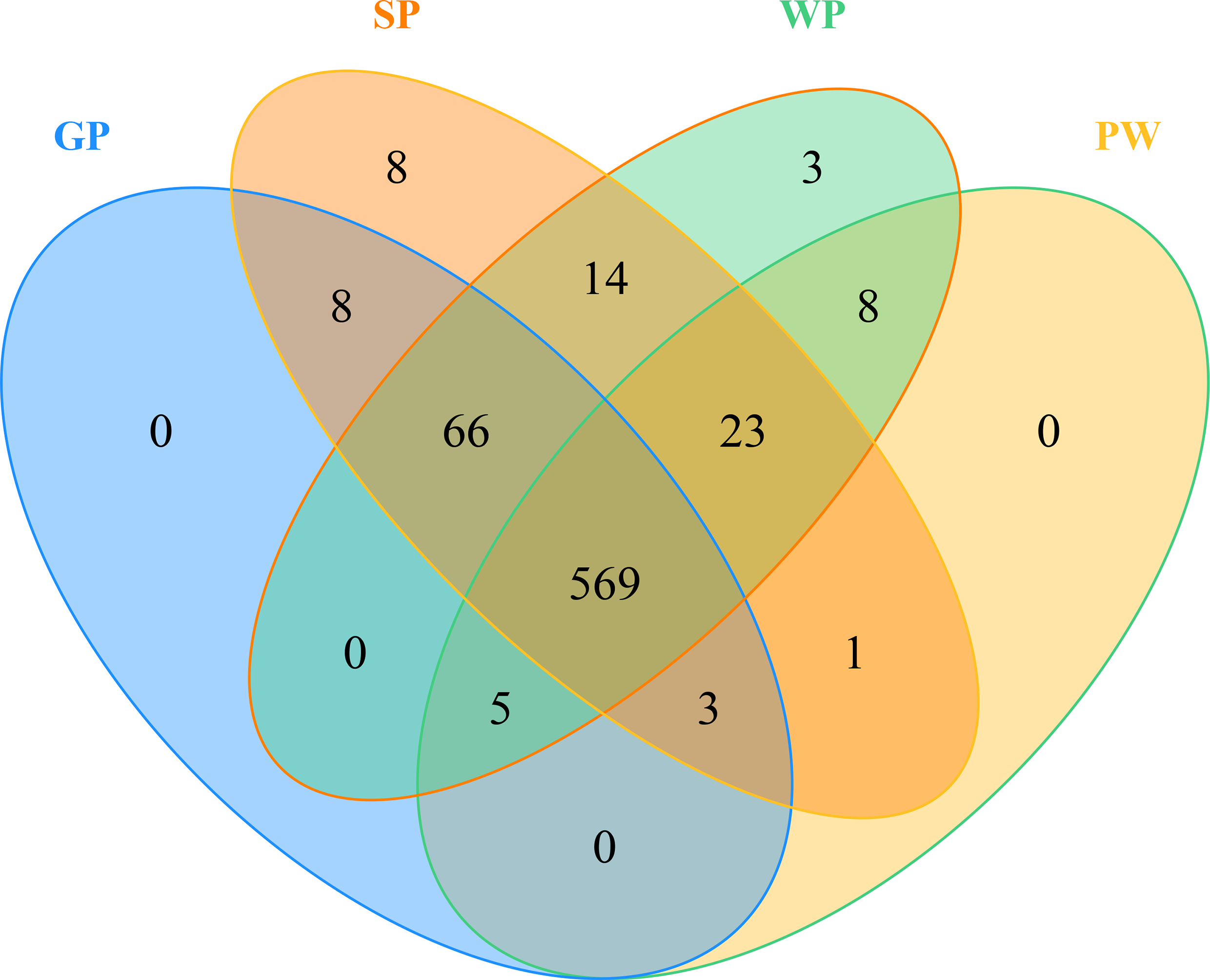

Supplement: Supplementary file 2 — Additional file 2: Fig. S1. Shared OTU analysis of the different groups. PW, fecal microbiota of the preweaning piglets; WP, fecal microbiota of the weaning piglets; GP, fecal microbiota of the growth pigs; SP, fecal microbiota of the sow. [file 13568_2021_1249_MOESM2_ESM.tif]

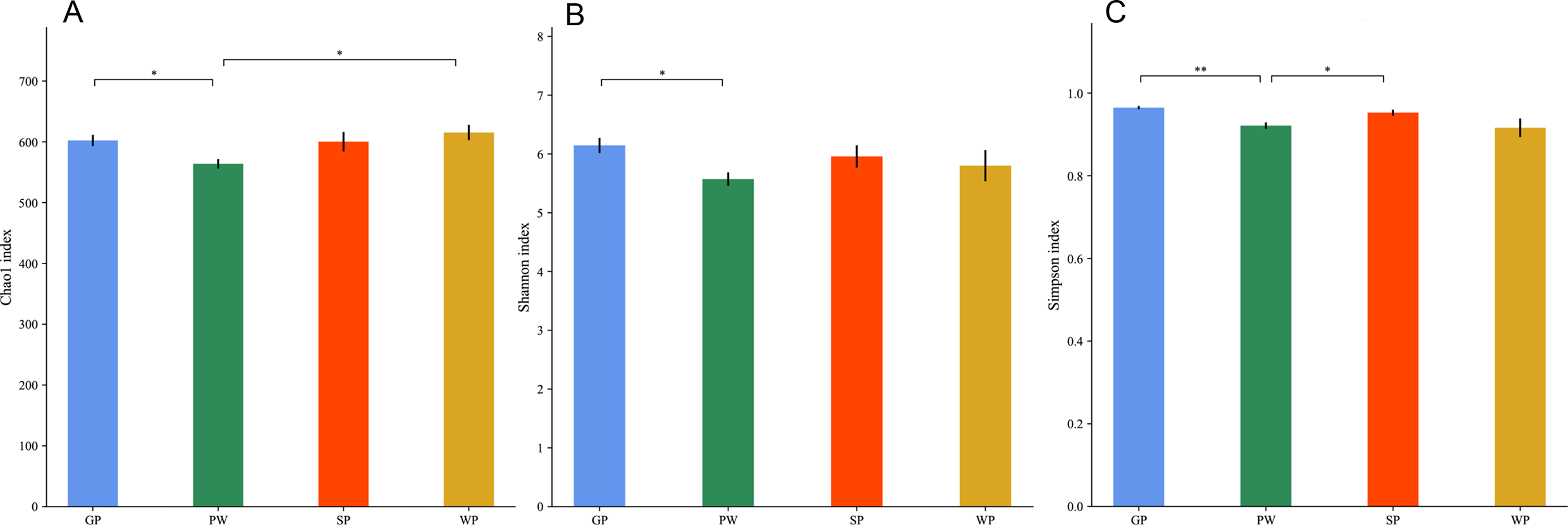

Supplement: Supplementary file 3 — Additional file 3: Fig. S2. Dynamic changes in intestinal microorganism alpha diversity. (A) Chao1 index; (B) Shannon index, (C) Simpson index. Different letters represent significant differences in alpha diversity indices based on Student’s t-test (* p < 0.05, ** p < 0.01). [file 13568_2021_1249_MOESM3_ESM.tif]

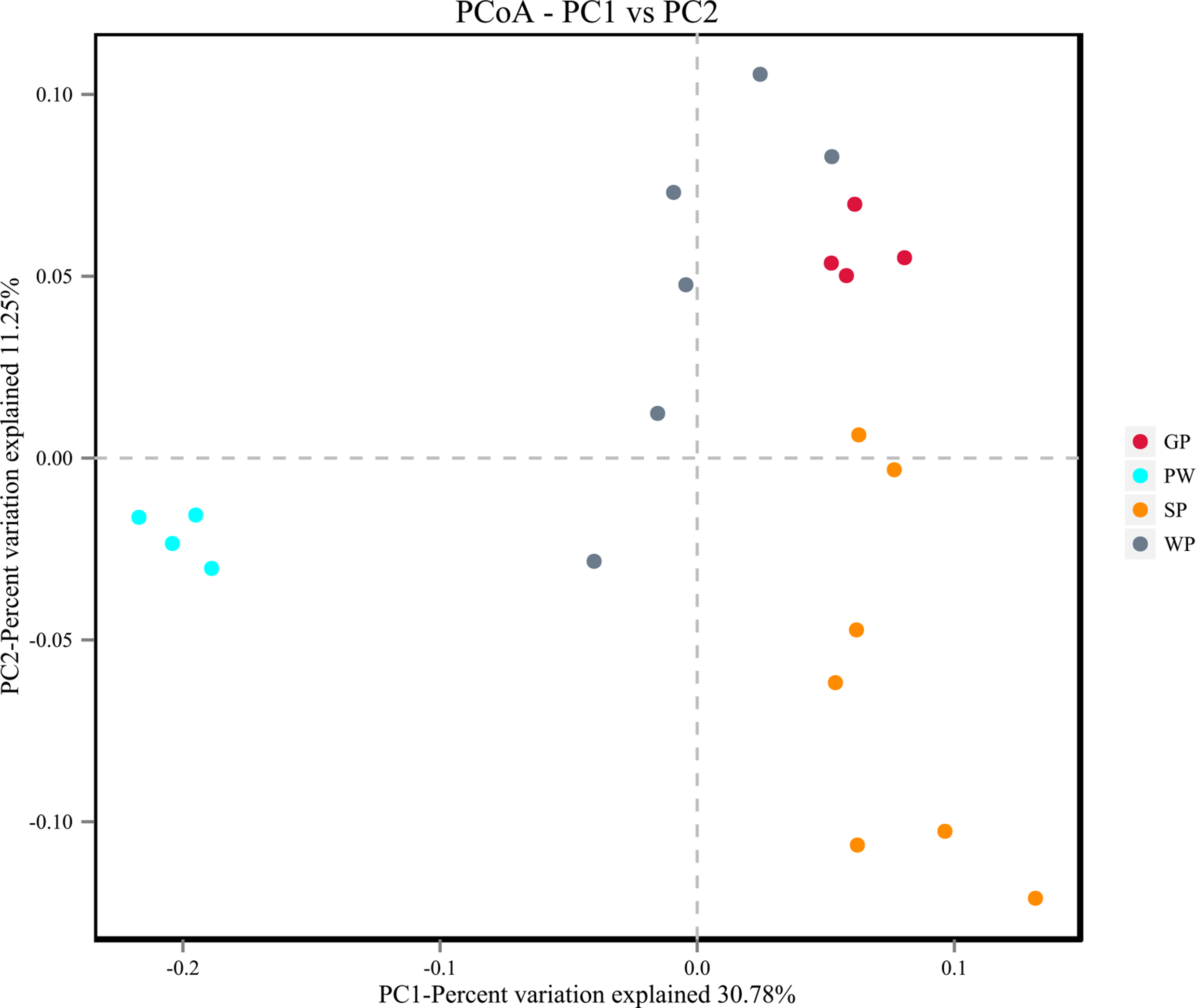

Supplement: Supplementary file 4 — Additional file 4: Fig. S3. Principal coordinates analysis (PCoA) in fecal microbiota of pigs at different grow stages. [file 13568_2021_1249_MOESM4_ESM.tif]

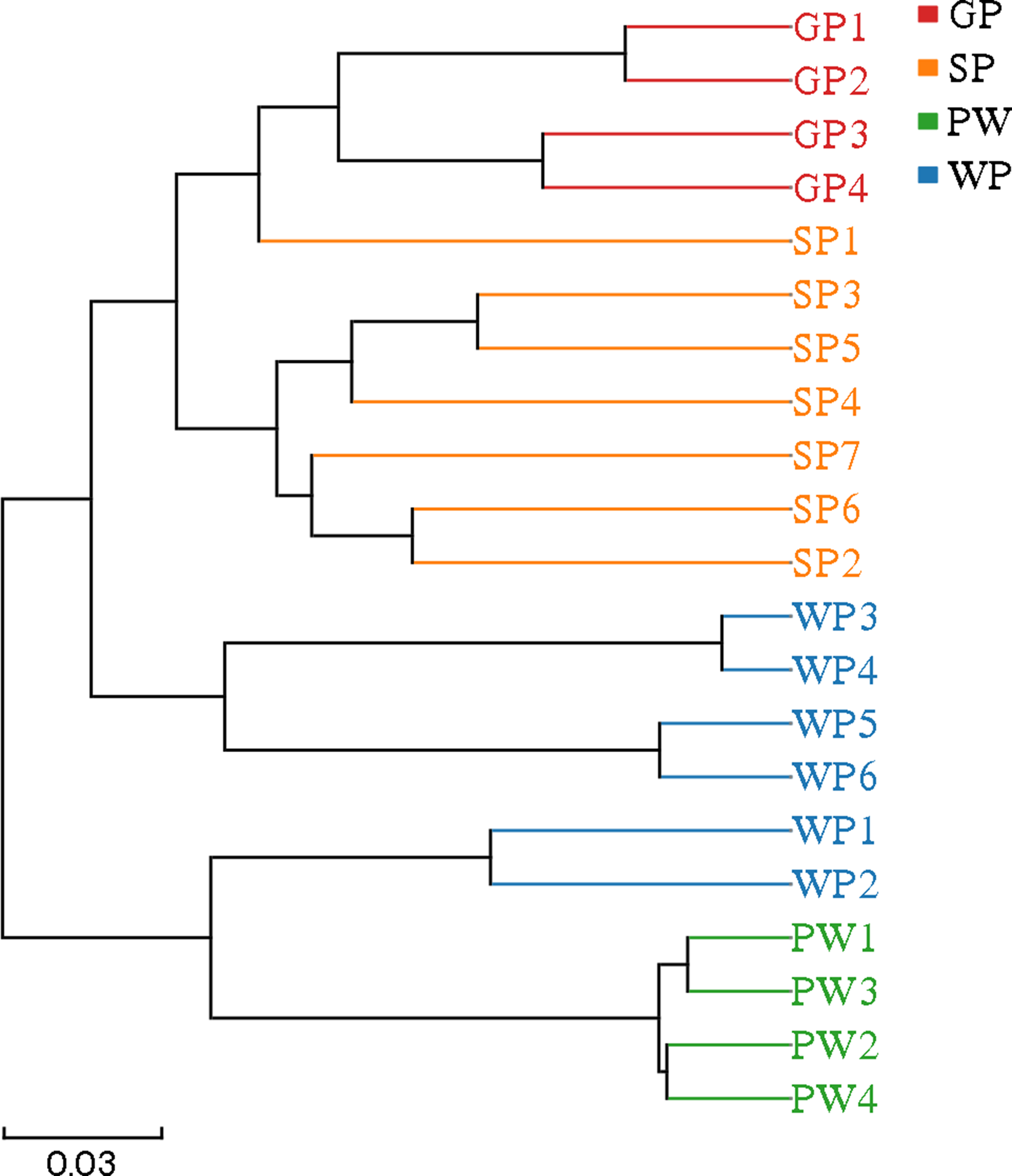

Supplement: Supplementary file 5 — Additional file 5: Fig. S4. Unweighted pair-group method with arithmetic mean (UPGMA) phylogenetic tree analysis. [file 13568_2021_1249_MOESM5_ESM.tif]

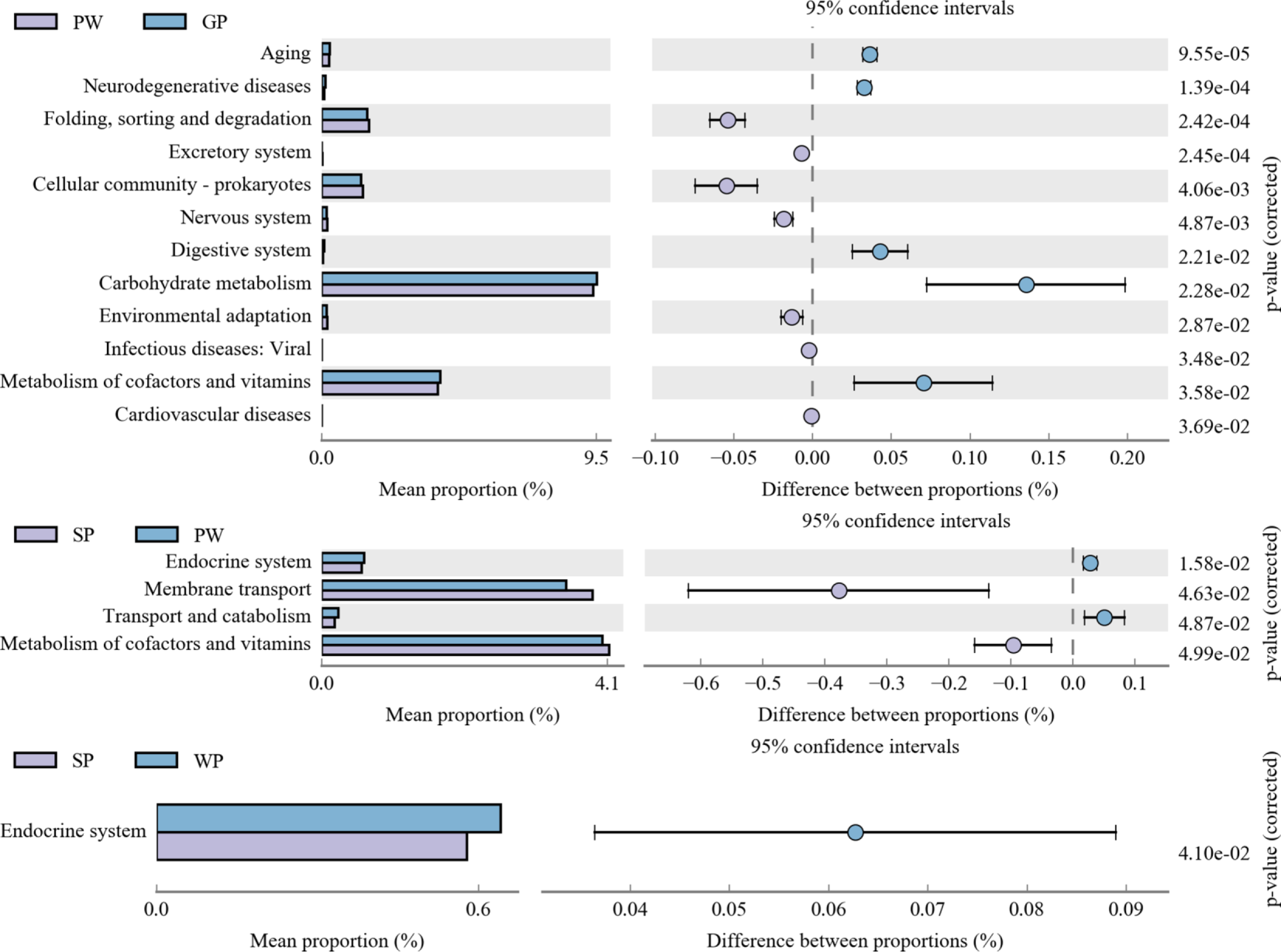

Supplement: Supplementary file 6 — Additional file 6: Fig. S5. Analysis of the difference of KEGG metabolic pathway between groups at the second level. Comparison between preweaning piglets and growth pigs, between preweaning piglets and sow, between weaning piglets and sow. The proportion of functional-abundance differences within the 95% confidence interval. [file 13568_2021_1249_MOESM6_ESM.tif]
